# Glycerol restores p53-dependent radiosensitivity of human head and neck cancer cells bearing mutant p53

**DOI:** 10.1054/bjoc.2000.1511

**Published:** 2000-12

**Authors:** K Ohnishi, I Ota, A Takahashi, T Ohnishi

**Affiliations:** Departments of Biology, Otorhinolaryngology, Nara Medical University, 840 Shijo-cho, Kashihara, Nara, 634-8521, Japan

**Keywords:** p53, apoptosis, Bax, radiosensitivity, glycerol, chemical chaperone

## Abstract

Mutation or inactivation of p53 is known to be present in approximately 50% of human cancers. We propose here a novel strategy for overcoming this problem in mutant p53-targeting cancer therapies. We examined the restoration of radiation-induced p53-dependent apoptosis by a chemical chaperone (glycerol) in human head and neck cancer cells (SAS cells, showing wild-type *p53* phenotype). SAS cells transfected with mutant *p53* (SAS/m *p53*) showed radioresistance compared with SAS cells (SAS/ *neo*) transfected with *neo* vector as a control, but became radiosensitive when pre-treated with glycerol before X-ray irradiation. Apoptosis in the SAS/m *p53* cells was induced by X-rays with glycerol pre-treatment, but not without glycerol pre-treatment, whereas apoptosis in the SAS/ *neo* cells was induced in both cases. Gel mobility-shift assays showed that after X-ray irradiation combined with glycerol pre-treatment, mp53 was able to bind to the sequence-specific region upstream of the *bax* gene regulating apoptosis. These results suggest that glycerol is effective in inducing a conformational change of p53 and restoring normal function to mp53, leading to enhanced radiosensitivity through the induction of apoptosis. This novel tool for enhancement of radiosensitivity in cancer cells bearing mp53 may be useful for p53-targeted radiotherapy. © 2000 Cancer Research Campaign http://www.bjcancer.com


*British Journal of Cancer* (2000) **83**(12), 1735–1739
© 2000 Cancer Research Campaign
doi: 10.1054/ bjoc.2000.1511, available online at http://www.idealibrary.com on IDEAL®
http://www.bjcancer.com

### Glycerol restores p53-dependent radiosensitivity of human head and neck cancer cells bearing mutant p53

**K Ohnishi, I Ota*, A Takahashi and T Ohnishi**

Departments of Biology and *Otorhinolaryngology, Nara Medical University, 840 Shijo-cho, Kashihara, Nara 634-8521, Japan

**Summary** Mutation or inactivation of p53 is known to be present in approximately 50% of human cancers. We propose here a novel strategy for overcoming this problem in mutant p53-targeting cancer therapies. We examined the restoration of radiation-induced p53-dependent apoptosis by a chemical chaperone (glycerol) in human head and neck cancer cells (SAS cells, showing wild-type *p53* phenotype). SAS cells transfected with mutant *p53* (SAS/m*p53*) showed radioresistance compared with SAS cells (SAS/*neo*) transfected with *neo* vector as a control, but became radiosensitive when pre-treated with glycerol before X-ray irradiation. Apoptosis in the SAS/m*p53* cells was induced by X-rays with glycerol pre-treatment, but not without glycerol pre-treatment, whereas apoptosis in the SAS/*neo* cells was induced in both cases. Gel mobility-shift assays showed that after X-ray irradiation combined with glycerol pre-treatment, mp53 was able to bind to the sequence-specific region upstream of the *bax* gene regulating apoptosis. These results suggest that glycerol is effective in inducing a conformational change of p53 and restoring normal function to mp53, leading to enhanced radiosensitivity through the induction of apoptosis. This novel tool for enhancement of radiosensitivity in cancer cells bearing mp53 may be useful for p53-targeted radiotherapy. © 2000 Cancer Research Campaign http://www.bjcancer.com

**Keywords**: p53; apoptosis; Bax; radiosensitivity; glycerol; chemical chaperone

Wild-type p53 (wt p53) has multi-functions, including suppression of cancer initiation and tumour growth through the induction of downstream genes and/or protein interaction (Caelles et al, 1994; Dulic et al, 1994; El-Deiry et al, 1994; Waga et al, 1994). In a recent clinical report, we showed that wt*p53* patients show higher survival rate after radiotherapy, resulting from Bax and Bcl-2 regulation, compared with mutated p 53 (m*p53*) patients (Harima et al, 1998). To trigger the multi-functions of p53, p53 is required to form the correct conformation which enables p53 to bind to specific DNA sequences regulating gene expression. Mutations in the *p53* gene cause conformational alterations in the p53 protein, and the majority of mp53 proteins can no longer regulate expression of downstream genes (Bargonetti et al, 1991; Kern et al, 1991). M*p53* genes are frequently observed in human cancer cells (Hollstein et al, 1991; Jia et al, 1997). Thus, a strategy for inducing mp53 to fold correctly could be useful for cancer therapy. We have recently reported that heat stress induces WAF1 expression only when p53 protein is wtp53 using a human glioblastoma cell line (A-172, wt*p53*) and its transfectants with an m*p53* vector (A-172/m*p53*) (Ohnishi et al, 1996, 1998). A-172 m*p53* cells abolished heat-induced WAF1 expression due to the dominant negative nature of the mp53 protein over endogenous wtp53 (Kern et al, 1992; Unger et al, 1992). However, heat-induced WAF1 accumulation was restored in these m*p53*-transfected cells when the cells were pre-treated with glycerol (Ohnishi et al, 1999a). Glycerol thus appeared to confer wtp53 function on mp53. The function of glycerol as a chemical chaperone has been reported elsewhere (Thomas et al, 1995; Welch and Brown, 1996). Glycerol seems to correct the conformation of some types of proteins which cause human diseases.

*Received 15 March 2000*
*Revised 7 August 2000*
*Accepted 13 August 2000*

*Correspondence to:* T Ohnishi

### MATERIALS AND METHODS

### Cells

Head and neck cell line of human squamous cell carcinoma, SAS cells (provided by JCRB, Tokyo, Japan) were cultured at 37˚C in Dulbecco's Modified Eagle medium containing 10% (v/v) fetal bovine serum, penicillin (50 U $ml^{-1}$), streptomycin (50 μg $ml^{-1}$) and kanamycin (50 μg $ml^{-1}$) (DMEM-10) in a conventional humidified 5% $CO_2$ incubator.

### Plasmids

SAS cells were transfected with plasmid pC53–248, which contains an m*p53* gene (codon 248, from Arg to Trp) which encodes a dominant negative mp53, or with the control vector pCMV-Neo-Bam. These plasmids were provided by B Vogelstein, Johns Hopkins Oncology Center, MD, USA. The stable transfectants SAS/m*p53* and SAS/*neo* were selected with G418 (200–400 μg $ml^{-1}$, Sigma Chemical Co, St Louis, MO), and used for the present experiments. Detailed procedure for transfection is described elsewhere (Ohnishi et al, 1998).

### Glycerol treatment

Cells were treated with glycerol (at final concentration of 0.6 M) 48 h before X-ray irradiation and then were incubated at 37˚C for 10 h in the presence of glycerol until sampling. X-ray irradiation was performed in the presence of glycerol. In the case of cell survival assay, the medium with glycerol was changed with glycerol-free one after 10 h incubation and thereafter cells were incubated for 10 to 14 days at 37°C in glycerol-free medium.

### X-ray irradiation

For the X-ray irradiation treatment, subconfluent cells in 25-cm$^2$ flasks containing DMEM-10 were exposed to X-rays (2.5–10 Gy) using a 150 kVp X-ray generator (Model MBR-1520R, Hitachi, Tokyo, Japan) with a total filtration of 0.5 mm aluminium plus 0.1 mm copper filter, and then incubated at 37°C.

### Cell survival assay

To measure radiosensitivity of the cells, cell survival after X-ray irradiation was quantitated by plating cells into 25 cm$^2$ flask containing the medium. Thereafter, cell colonies were rinsed with PBS, fixed with methanol, stained with 2% Giemsa solution (Merck, Woodbridge, NJ, USA). Colonies containing at least 50 cells were counted. The number of cells per colony was determined prior to experiments.

### Analysis of apoptosis

Induction of apoptosis was analysed by detection of both DNA fragmentation and apoptotic bodies. To detect DNA fragmentation in the nucleosomal size range, cells were suspended in lysis buffer containing 10 mM Tris-HCl, pH 7.4, 10 mM NaCl, 10 mM EDTA and 1% sodium N-lauroylsarcosinate, incubated with 100 μg ml$^{-1}$ proteinase K at 37°C overnight, and then centrifuged at 18500 ***g*** for 30 min. The resulting supernatants were incubated with 100 μg ml$^{-1}$ RNase A at 37°C for 1 h. The DNA in the solution was precipitated with ethanol at –20°C, dissolved in 10 mM Tris-HCl, pH 8.0, and 1 mM EDTA, and electrophoresed for 30 min at 100 V through 3% NuSieve 3:1 gels containing 40 mM Tris-acetate, pH 7.8, 2 mM EDTA and 0.5 μg ml$^{-1}$ ethidium bromide. After electrophoresis, the gels were photographed under ultraviolet light. For detection of apoptotic bodies, cells were fixed with 1% glutaraldehyde (Nakalai Tesque, Kyoto, Japan) in PBS at 4°C, washed with PBS, stained with 0.2 mM Hoechst 33342 (Sigma Chemical Co), and then observed under a fluorescence microscope. At least 300 cells were counted in each experiment.

### Gel mobility-shift assay

In in vivo samples, the cells (about 2 × 10$^7$ cells) were irradiated with X-rays (6 Gy) in the presence or absence of glycerol (0.6 M). The nuclear extracts were prepared 6 h after X-ray irradiation according to the method described elsewhere (Ohnishi et al, 1998). In in vitro samples, whole cell extracts were prepared from intact cells (about 2 × 10$^7$ cells) and treated with glycerol (0.6 M), X-rays (6 Gy) or a combination of glycerol and X-rays (6 Gy), and subsequently incubated for 30 min at 37°C. The p53–p53 consensus sequence (p53CON) binding activity was measured by a gel mobility-shift assay (Ohnishi et al, 1998) using a synthetic double-stranded DNA fragment encoding the p53CON (5′-GGACATGC-CCGGGCATGTCC-3′, Japan Bioservice, Niiza, Saitama, Japan) based on a specific sequence located upstream of the *bax* gene as a probe.

### RESULTS AND DISCUSSION

To examine the effect of glycerol on the radiosensitivity of SAS/m*p53* and SAS/*neo* cells, the clonogenic surviving fractions after X-ray irradiation combined with pre-treatment with or without glycerol (0.6 M) were measured. As shown in Figure 1, the SAS/m*p53* cells were about 1.5 times more resistant to X-rays than the SAS/*neo* cells at the $D_{10}$ dose. However, when pre-treated with glycerol, SAS/m*p53* cells become about 1.5 times more X-ray-sensitive at the $D_{10}$ dose. Glycerol caused little enhancement of X-ray sensitivity in SAS/*neo* cells. On the other hand, SAS/m*p53* and SAS/*neo* cells treated with glycerol alone (0.6 M) showed about 80% surviving fractions, and thus 0.6 M glycerol appeared to cause no serious cell damage regardless of *p53* status. These results suggest that glycerol affects the radiosensitivity of these cells in a p53-dependent manner.

Furthermore, we examined the induction of apoptosis by detection of apoptotic bodies and DNA fragmentation to elucidate the mechanism of enhancement of radiosensitivity by glycerol in SAS/m*p53* cells. We analysed the nuclear morphology with Hoechst 33342 staining. We scored the number of apoptotic bodies such as those shown by arrows in the insert of Figure 2. The incidence of apoptosis increased with time after X-ray irradiation (6 Gy) in SAS/*neo* cells (about 10% at 48 h), while there was almost no increase in the incidence of apoptosis up to 72 h after

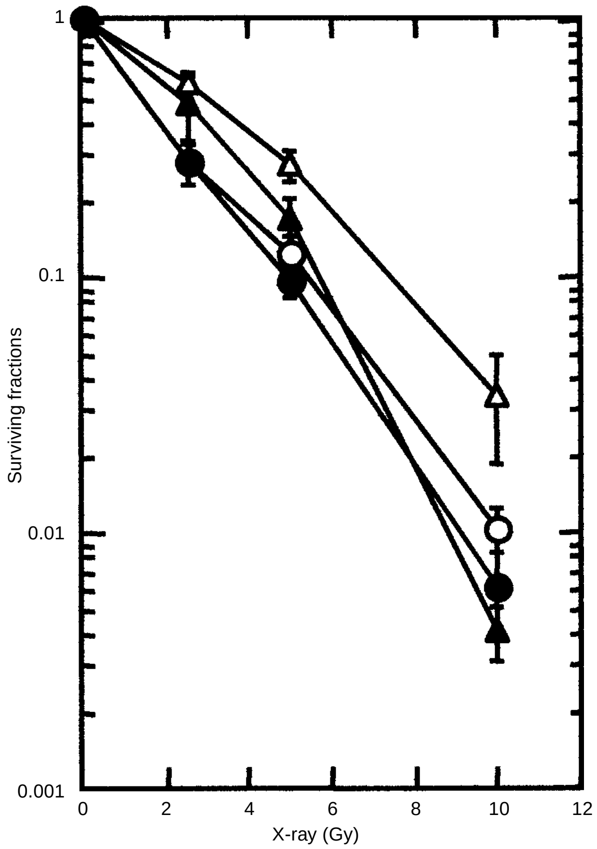

**Figure 1** Clonogenic surviving fractions of transformant SAS cells after X-ray irradiation measured by the colony formation assay. Open circles, SAS/*neo* cells; closed circles, SAS/*neo* cells pre-treated with 0.6 M glycerol for 48 h before X-ray irradiation; open triangles, SAS/m*p53* cells; closed triangles, SAS/m*p53* cells pre-treated with 0.6 M glycerol. Surviving fractions were measured in 3 independent duplicate experiments

 © 2000 Cancer Research Campaign

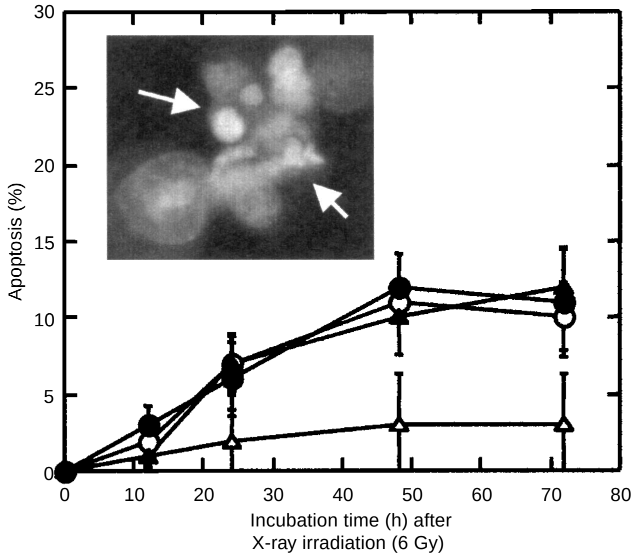

**Figure 2** Apoptotic bodies detected with Hoechst 33342 staining. The cells were incubated at 37˚C for 0, 12, 24, 48 or 72 h after X-ray irradiation (6 Gy) with or without glycerol (0.6 M), fixed with glutaraldehyde, stained with Hoechst 33342, and then observed under a fluorescence microscope. Apoptotic bodies were counted under 3 different fields of the microscopic observation. 100 cells were judged under one field. The symbols are the same as in Figure 1. Inserted photograph, SAS/m*p53* cells 48 h after X-ray irradiation (6 Gy) with glycerol (0.6 M) treatment. Arrows indicate apoptotic bodies

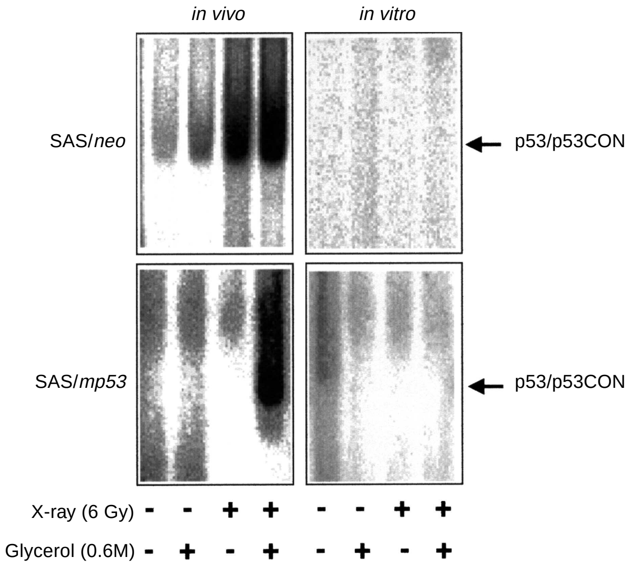

**Figure 4** DNA binding activity of nuclear or whole cell extracts from SAS/*neo* or SAS/m*p53* cells for p53CON. In in vivo sample, the cells were incubated at 37˚C for 6 h after glycerol (0.6 M) treatment or X-ray irradiation (6 Gy) combined with or without glycerol. After the incubation, the cells were washed with PBS and harvested for gel mobility-shift assay. In in vitro sample, whole cell extracts from SAS/*neo* or SAS/m*p53* cells were treated with glycerol (0.6 M) or X-ray irradiation (6 Gy) combined with or without glycerol and then incubated for 30 min at 37˚C. The band position of the specific p53-p53CON complex is shown by arrow

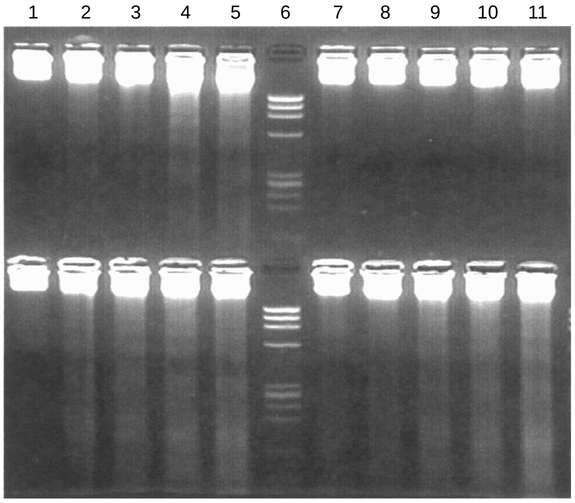

**Figure 3** DNA fragmentation detected by agarose gel electrophoresis of DNA extracted from SAS/*neo* (lanes 1–5) and SAS/m*p53* cells (lanes 7–11) after X-ray irradiation (6 Gy) (upper panel) or after X-ray irradiation (6 Gy) in the presence of 0.6 M glycerol (lower panel). After the incubation at 37˚C for 12, 24, 42, or 72 h, the cells were suspended in the lysis buffer to extract DNA for gel electrophoresis. Lanes 1 and 7, unirradiated; lanes 2 and 8, at 12 h; lanes 3 and 9, at 24 h; lanes 4 and 10, at 42 h; lanes 5 and 11, at 72 h after X-ray irradiation. Lane 6, øX174 DNA *Hae* III fragments as size markers

X-ray irradiation in SAS/m*p53* cells (about 3%). The rate of apoptosis in SAS/*neo* cells was about 5-fold higher than that in SAS/m*p53* cells at 48–72h after X-ray irradiation (Figure 2). When treated with glycerol (0.6 M) before X-ray irradiation, the SAS/m*p53* cells underwent apoptosis with time after X-ray irradiation (6 Gy). The time course of X-ray-induced apoptosis in the glycerol-treated SAS/m*p53* cells was similar to that in SAS/*neo* cells. We next examined the induction of DNA fragmentation after X-ray irradiation (6 Gy) using agarose gel electrophoresis of DNA extracted from SAS/*neo* (Figure 3, lanes 1–5) and SAS/m*p53* cells (Figure 3, lanes 7–11). No DNA ladder formation was detected after X-ray irradiation alone in SAS/m*p53* cells (Figure 3, upper right panel). However, some DNA ladders in the nucleosomal size range were observed 24–72 h after X-ray irradiation combined with glycerol (0.6 M) (Figure 3, lower right panel). In contrast, SAS/*neo* showed the DNA ladders after X-ray irradiation in the absence (Figure 3, upper left panel) or presence (Figure 3, lower left panel) of 0.6 M glycerol. Glycerol alone induced no apoptotic bodies or DNA fragmentation in either type of transformed cells (data not shown). These results showed that the X-ray-induced apoptosis was p53-dependent.

To confirm whether glycerol induces mp53 to adapt the conformation of wtp53, the DNA binding activity of p53 for p53CON was measured in nuclear or whole cell proteins extracted from SAS/*neo* or SAS/m*p53* cells using the gel mobility-shift assay (Figure 4). Wt p53 is known to bind to p53CON, which is homologous to a specific DNA sequence located upstream of the *bax* gene and which positively controls apoptosis (Miyashita and Reed, 1995). As supporting the evidence, in in vivo samples, DNA binding activity of nuclear proteins was increased in SAS/*neo* cells treated with X-ray (6 Gy) combined with or without 0.6 M glycerol (Figure 4). Mp53 can not enhance apoptosis, because most mp53 can not bind to the specific DNA sequence. In fact, in in vivo samples, no DNA binding activity of nuclear proteins was detected in the intact SAS/m*p53* cells (Figure 4, lane 1 of in vivo) and this activity level was unchanged even when SAS/m*p53* cells were treated with glycerol (0.6 M) or X-rays (6 Gy) (Figure 4,

*© 2000 Cancer Research Campaign*

lanes 2 and 3 of in vivo). The defective DNA binding ability of p53 from SAS/m*p53* cells may be due to the dominant negative nature of this mp53 protein (Kern et al, 1992; Unger et al, 1992). In contrast, when SAS/m*p53* cells were treated with glycerol (0.6 M) before X-ray irradiation, nuclear proteins prepared from the cells showed a clear increase of DNA binding activity, probably due to binding of p53 to p53CON (Figure 4 lane 4 of in vivo). When unlabelled p53CON probe ($\times$ 100) was added to the reaction mixture containing $^{32}$P-labelled p53CON and the nuclear proteins from SAS/m*p53* cells treated with X-rays combined with glycerol, the binding of p53 to p53CON disappeared (data not shown). This indicates that the observed bands are p53-p53CON specific bands. From these results, the apoptosis observed in SAS/m*p53* cells (Figures 2 and 3) might be induced through *bax* gene expression which is up-regulated by the activated mp53, which underwent a conformational change to wtp53 as reported elsewhere (Brown et al, 1997). The acquisition of binding activity of glycerol-treated mp53 is likely to demand cellular signal transduction induced by X-rays, because no binding activity of p53 was observed when whole cell proteins extracted from intact SAS/*neo* or SAS/m*p53* cells were treated with X-ray (Figure 4, lane 3 of in vitro) or X-ray combined with glycerol (Figure 4, lane 4 of in vitro).

We showed that X-ray or glycerol treatment alone was insufficient to convert the conformation of mp53 to that of active wtp53, because neither treatment led to an increase in the DNA binding activity of p53 (Figure 4). Therefore, we assume that conformational change of mp53 to that of wtp53 induced by glycerol is not sufficient to trigger apoptosis, and some initial signals evoked by X-ray irradiation are required for effective signals leading to apoptosis. Conformation-stabilized p53 produced by glycerol treatment is probably activated through phosphorylation by X-ray-activated protein kinases such as ATM (Banin et al, 1998; Canman et al, 1998; Khanna et al, 1998; Nakagawa et al, 1999), ATR (Tibbetts et al, 1999), PKC (Takenaka et al, 1995) or other kinases (Hall et al, 1996, Jamal and Ziff, 1995). The present results suggest at least that the apoptosis induced by X-rays reported here depends on p53-dependent signal transduction. Glycerol may be generally effective against p53-regulated downstream genes through the restoration of the function of mp53, because restoration of mp53 function to normal by glycerol is observed in WAF1 induction as well (Ohnishi et al, 1999a).

Possible other explanations can be proposed for the increased DNA binding activity of nuclear extracts from SAS/m*p53* cells after combined X-ray and glycerol treatments. Overexpressed mp53 may obtain the ability to enhance the DNA binding activity of endogenous wtp53 due to reactions triggered by the combined treatments. Unknown signal transduction pathways may contribute to the modification of mp53 function.

Some investigators have vigorously searched for molecules which have an ability to induce normal function of p53 in inactive p53 or mp53. The latent sequence-specific DNA-binding function is activated by small peptides (Hupp et al, 1995). As already described, glycerol induces conformational change of mp53 to wtp53 (Brown et al, 1997) and restores the ability of mp53 to induce WAF1 expression (Ohnishi, 1999a,b). Similar chaperone-like function has been also found in other molecules such as a synthetic peptide derived from p53 C-terminal domain (Selivanova et al, 1997) and a small compound (Foster et al, 1999). Such manipulation of mp53 or inactive p53 by these molecules may provide new cancer therapies effective for patients carrying mp53 tumours.

### ACKNOWLEDGEMENTS

This work was supported by Grants-in-Aid from the Ministry of Education, Science, Sports and Culture of Japan.

### REFERENCES

Banin S, Moyal L, Shieh S, Taya Y, Anderson CW, Chessa L, Smorodinsky NI, Prives C, Reiss Y, Shiloh Y and Ziv Y (1998) Enhanced phosphorylation of p53 by ATM in response to DNA damage. *Science* **281**: 1674–1677

Bargonetti J, Friedman PN, Kern SE, Vogelstein B and Prives C (1991) Wild-type but not mutant p53 immunopurified proteins bind to sequences adjacent to the SV40 origin of replication. *Cell* **65**: 1083–1091

Brown CR, Hong-Brown LQ and Welch WJ (1997) Correctiong temperature-sensitive protein folding defects. *J Clin Invest* **99**: 1432–1444

Caelles C, Helmberg A and Karin M (1994) p53-dependent apoptosis in the absence of transcriptional activation of p53-target genes. *Nature* **370**: 220–223

Canman CE, Lim DS, Cimprich KA, Taya Y, Tamai K, Sakaguchi K, Appella E, Kastan MB and Siliciano JD (1998) Activation of the ATM kinase by ionizing radiation and phosphorylation of p53. *Science* **281**: 1677–1679

Dulic V, Kaufmann WK, Wilson SJ, Tlsty TD, Lees E, Harper JW, Elledge SJ and Reed SI (1994) p53-dependent inhibition of cyclin-dependent kinase activities in human fibroblasts during radiation-induced $G_1$ arrest. *Cell* **76**: 1013–1023

El-Deiry WS, Harper JW, O'Connor PM, Velculescu VE, Canman CE, Jackman J, Pietenpol JA, Burrell M, Hill DE, Wang Y, Wilman KG, Mercer WE, Kastan MB, Kohn KW, Elledge SJ, Kinzler KW and Vogelstein B (1994) *WAF1/CIP1* is induced in *p53*-mediated $G_1$ arrest and apoptosis. *Cancer Res* **54**: 1169–1174

Foster BA, Coffey HA, Morin MJ and Rastinejad F (1999) Pharmacological rescue of mutant p53 conformation and function. *Science* **286**: 2507–2510

Hall SR, Campbell LE and Meek DW (1996) Phosphorylation of p53 at the casein kinase II site selectively regulates p53-dependent transcriptional repression but not transactivation. *Nucleic Acids Res* **24**: 1119–1126

Harima Y, Harima K, Shikata N, Oka A, Ohnishi T and Tanaka Y (1998) Bax and Bcl-2 expressions predict response to radiotherapy in human cervical cancer. *J Cancer Res Clin Oncol* **124**: 503–510

Hollstein M, Sidransky D, Vogelstein B and Hallis CC (1991) p53 mutations in human cancers. *Science* **253**: 49–53

Hupp TR, Sparks A and Lane DP (1995) Small peptides activate the latent sequence-specific DNA binding function of p53. *Cell* **83**: 237–245

Jamal S and Ziff EB (1995) Raf phosphorylates p53 *in vitro* and potentiates p53-dependent transcriptional transactivation *in vivo*. *Oncogene* **10**: 2095–2101

Jia LQ, Osada M and Ishioka C, Gamo M, Ikawa S, Suzuki T, Shimodaira H, Niitani T, Kudo T, Akiyama M, Kimura N, Matsuo M, Mizusawa H, Tanaka N, Koyama H, Wamba M, Kanamura R and Kuroki T (1997) Screening the p53 status of human cell lines using a yeast functional assay. *Mol Carcinog* **19**: 243–253

Kern SE, Kinzler KW, Bruskin A, Jarosz D, Friedman P, Prives C and Vogelstein B (1991) Identification of p53 as a sequence-specific DNA-binding protein. *Science* **252**: 1708–1711

Kern SE, Pietenpol JA, Thiagalingam S, Seymour A. Kinzler KW and Vogelstein B (1992) Oncogenic forms of p53 inhibit p53-regulated gene expression. *Science* **256**: 827–830

Khanna KK, Keating KE, Kozlov S, Scott S, Gatei M, Hobson K, Taya Y, Gabrielli B, Chan D, Lees-Miller SP and Lavin MF (1998) ATM associates with and phosphorylates p53: mapping the region of interaction. *Nat Genet* **20**: 398–400

Miyashita T and Reed JC (1995) Tumor suppressor p53 is a direct transcriptional activator of the human *bax* gene. *Cell* **80**: 293–299

Nakagawa K, Taya Y, Tamai K and Yamaizumi M (1999) Requirement of ATM in phosphorylation of the human p53 protein at serine 15 following DNA double-strand breaks. *Mol Cell Biol* **19**: 2828–2834

Ohnishi T, Wang X, Ohnishi K, Matsumoto H and Takahashi A (1996) p53-dependent induction of WAF1 by heat treatment in human glioblastoma cells. *J Biol Chem* **271**: 14510–14513

Ohnishi K, Wang X, Takahashi A and Ohnishi T (1998) Contribution of protein kinase C to p53 dependent WAF1 induction pathway after heat treatment in human glioblastoma cell lines. *Exp Cell Res* **238**: 399–406

Ohnishi T, Ohnishi K, Wang X, Takahashi A and Okaichi K (1999a) Restoration of mutant TP53 to normal TP53 function by glycerol as a chemical chaperone. *Radiat Res* **151**: 498–500

Ohnishi T, Matsumoto H, Wang X, Takahashi A and Ohnishi K (1999b) Restoration of p53-dependent apoptosis in the cells bearing the mutant *p53* gene by glycerol. *Int J Radiat Biol* **75**: 1095–1098

*© 2000 Cancer Research Campaign*

Selivanova G, Iotsova V, Okan I, Fritsche M, Strom M, Groner B, Grafstrom RC and Wiman KG (1997) Restoration of the growth suppression function of mutant p53 by a synthetic peptide derived from the p53 C-terminal domain. *Nat Med* **3**: 632–638

Takenaka I, Morin F, Seizinger BR and Kley N (1995) Regulation of the sequence-specific DNA binding function of p53 by protein kinase C and protein phosphatases. *J Biol Chem* **270**: 5405–5411

Thomas PJ, Qu BH and Pedersen PL (1995) Defective protein folding as a basis of human disease. *TIBS* **20**: 456–459

Tibbetts RS, Brumbaugh KM, Williams JM, Sarkaria JN, Cliby WA, Shieh SY, Taya Y, Prives C and Abraham RT (1999) A role for ATR in the DNA damage-induced phosphorylation of p53. *Genes Dev* **13**: 152–157

Unger T, Nau MM, Segal S and Minna JD (1992) p53: a transdominant regulator of transcription whose function is ablated by mutations occurring in human cancer. *EMBO J* **11**: 1383–1390

Waga S, Hannon GJ, Beach D and Stillman B (1994) The p21 inhibitor of cyclin-dependent kinases controls DNA replication by interaction with PCNA. *Nature* **369**: 574–577

Welch WJ and Brown CR (1996) Influence of molecular and chemical chaperones on protein folding. *Cell Stress Chap* **1**: 109–115

*© 2000 Cancer Research Campaign*
